# Optimal Correction of Adult Spinal Deformities Requires Restoration of Distal Lumbar Lordosis

**DOI:** 10.1155/2021/5572181

**Published:** 2021-05-06

**Authors:** S. Pesenti, S. Prost, A. Muñoz McCausland, K. Farah, P. Tropiano, S. Fuentes, B. Blondel

**Affiliations:** Aix-Marseille Université, APHM, CNRS, ISM, Hôpital de La Timone, Spine Unit 264 Rue Saint Pierre, Marseille 13005, France

## Abstract

**Purpose:**

The aim of this study is to analyze results according to postoperative pelvic incidence-lumbar lordosis (PI-LL) mismatch in the management of adult spine deformity (ASD) patients. Recently, it has been reported that in addition to lumbar lordosis amount, lordosis repartition between its proximal and distal parts was crucial.

**Methods:**

We enrolled 77 consecutive ASD patients who underwent posterior spinal fusion and deformity correction between 2015 and 2018. On preoperative and 1-year follow-up radiographs, we analyzed different parameters such as L1-S1 lumbar lordosis, L1-L4 proximal lordosis (PLL), L4-S1 distal lordosis (DLL), pelvic tilt (PT), sagittal vertical axis (SVA), and PI-LL mismatch. Comparisons were performed according to postoperative PI-LL mismatch (defined as “aligned” when PI-LL was <10°). The relationship between lordosis distribution and postoperative alignment status was investigated.

**Results:**

On the whole series, average lumbar lordosis, SVA, and PI-LL improved (28.2° vs.43.5°, 82 vs. 51 mm, and 26°vs. 14°, all *p* < 0.001, respectively). On the other hand, PT remained unchanged (30° vs. 28°, *p* > 0.05). 35 patients were classified as “aligned” and 42 as “not aligned.” Patients from the “aligned” group had a significantly lower PI than patients from the “not aligned” group (52° vs. 61°, *p*=0.009). Postoperative PLL was not different between groups (18° vs. 16° *p* > 0.05), whereas DLL was significantly higher in the “aligned” group (31° vs. 22°, *p*=0.003). PI-LL was significantly correlated to DLL (rho = 0.407, *p* < 0.001) but not with PLL (rho = 0.110, *p*=0.342).

**Conclusions:**

Our results revealed that in ASD patients, postoperative malalignment was associated with a lack of DLL restoration. “Not aligned” patients had also a significantly higher pelvic incidence. Specific attention must be paid to restore optimal distal lumbar lordosis in order to set the amount and the distribution of optimal postoperative lumbar lordosis.

## 1. Introduction

Adult spinal deformity covers a broad range of pathologies and can be responsible for disability and altered quality of life [1]. When a surgical procedure is indicated, the aim is to restore sagittal and coronal alignment. Since the last decade, it has been largely reported that correction of sagittal alignment was of primary importance and associated with better outcomes [2–4].

ASD is commonly described with the SRS-Schwab classification [5] that also serves as guideline for sagittal correction (SVA < 50 mm, PI-LL < 10°, and PT < 20°). However, despite recent advances such as age-related spinopelvic alignment thresholds [6], postoperative sagittal objectives are reached in less than 50% of the cases [7].

The key parameter to evaluate postoperative alignment is the PI-LL mismatch. This parameter has been reported as a predictor of success and is correlated with health-related quality of life scores [8]. However, taking into account only the L1-S1 lumbar lordosis (LL) can be misleading for two reasons [8]:

  The L4-S1 DLL was reported to be almost constant
  Pelvic incidence is significantly related to the L1-L4 PLL

It seems therefore crucial to evaluate not only the global amount of LL restoration but also the PI-LL mismatch according to PLL and DLL.

This study aimed to analyze the results of ASD surgical management according to postoperative PI-LL mismatch with the hypothesis that aligned patients had a better restoration of the DLL.

## 2. Methods

### 2.1. Study Design and Patient Population

After institutional review board approval (IRB 00009118), we conducted a retrospective analysis of a prospectively maintained database of ASD patients operated between 2015 and 2018. Prior to inclusion, every patient signed an informed consent.

Inclusion criteria were as follows: all adult patients managed surgically for ASD with a posterior fixation that included all the lumbar spine (upper level instrumented vertebra L1 or above, lower instrumented vertebra S1 or below), primary surgery or revision case, pre and postoperative full spine X-rays available, and one-year minimal follow-up (including full spine X-rays).

For each patient, demographic data, clinical scores (lumbar VAS and Oswestry Disability Index), and full spine X-rays (AP and lateral) were obtained preoperatively, in the immediate postoperative period, and at one-year of follow-up. Complications during the follow-up period were systematically noted.

The surgical procedure consisted of a posterior fusion and bone resection if required (Grade 2 or 3 of the Schwab classification [9]).

### 2.2. Radiographic Parameters

On lateral radiographs, the following parameters were measured: T4-T12 thoracic kyphosis (TK), L1-S1 lumbar lordosis, L1-L4 PLL, L4-S1 DLL, SVA, pelvic parameters (PI and PT), and mismatch between pelvic incidence and lumbar lordosis (PI-LL).

### 2.3. Statistical Analysis

Data were formulated as means and standard deviations. Comparisons were carried out using Student's *t*-test for normally distributed variables. The population was analyzed as a whole and then stratified according to the 1-year postoperative PI-LL mismatch. Patients were classified as the “aligned” group when postoperative PI-LL was below 10° and “not aligned” otherwise. The two groups were compared with regards to radiographic parameters and mechanical complications. The relationship between PI-LL mismatch and lumbar lordosis distribution was investigated using Spearman correlation tests. The significance level was set at 95% (i.e., *p* < 0.05).

## 3. Results

### 3.1. Population and Surgical Data

Among the 97 patients who underwent ASD surgical correction in our institution during the inclusion period, 77 patients met inclusion criteria for the present study. Fifty-six women and 21 men with a mean age of 66.5 years old (SD = 8.8) were included. On average, 14 levels were fused during the procedure (SD = 3), and lower instrumented level included iliac screws for 43 patients and S1 screws for 34 patients.

### 3.2. Global Radiographic Analysis

On the whole series, the mean preoperative PI was 54.3° SD = 13 and did not change during follow-up.

With regards to regional parameters, a significant improvement of LL and TK was noted between preoperative and last follow-up evaluation (28.2° vs. 43.5°, *p* < 0.001 and 30.2° vs. 43.9°, *p* < 0.001, respectively).

SVA and PI-LL mismatch were significantly reduced (81.9 mm vs. 50.9 mm, *p* < 0.001 and 26° vs. 13.5°, *p* < 0.001, respectively). On the other hand, PT remained unchanged between pre and 1-year assessment (29.9° vs. 28.1°, *p* > 0.05).

### 3.3. Aligned vs. Nonaligned Group Analysis

**Figure 1:**
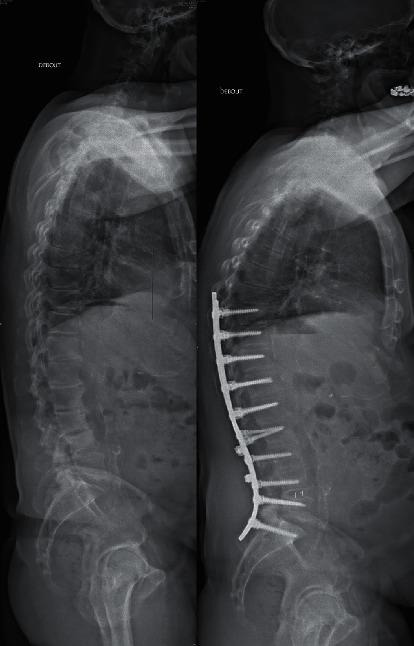
Clinical example of a patient from the “aligned group” with a low PI. Primary case, T9-S1 posterior fixation, multiples grade 2 osteotomies. Preoperative measurements (left) were PI = 41°, PT = 21°, PI-LL = 21, LL = -20°, TK = 22°, and SVA = 42 mm. One-year measurements (right) were PT = 17°, PI-LL = 0, LL = −41°, PLL = −8°, DLL = −33°, TK = 41°, and SVA = 41 mm.

**Figure 2:**
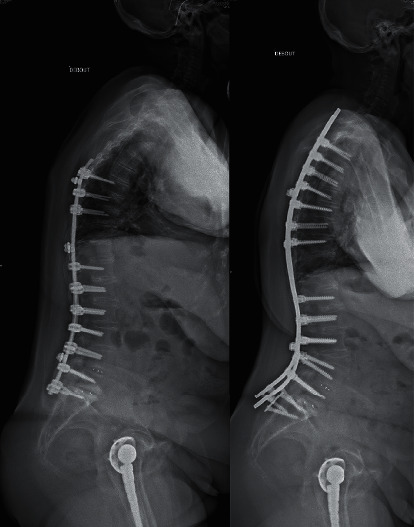
Clinical example of a patient form the “not aligned” group with a high PI. Revision case, T3-S2 posterior fixation, L3 grade 3 osteotomy. Preoperative measurements (left) were PI = 59°, PT = 34°, PI-LL = 32°, LL = −27°, TK = 3°, and SVA = 93 mm. One-year measurements (right) were PT = 25°, PI-LL = 15°, LL = −44°, PLL = −29°, DLL = −15°, TK = 36°, and SVA = 91 mm.

Thirty-five patients were classified as “aligned” (PI-LL <10°) and 42 were classified as “not aligned” (PI-LL >10°). Of the 42 “not aligned” patients, 17 were revision cases, 10 requiring a grade 3 osteotomy (8 in L3 and 2 in L4). Among the 35 “aligned” patients, 19 were revision cases, 7 requiring a grade 3 osteotomy (5 in L3 and 2 in L4) (Figures 1 and 2).

Preoperatively, “not aligned” patients had a significantly higher pelvic incidence, higher pelvic tilt, smaller lumbar lordosis, and thoracic kyphosis, despite a nonsignificantly different sagittal vertical axis (Table 1).

With regards to preoperative LL distribution, the L1-L4 PLL (1.5° vs. 08°, *p*=0.9) and the L4-S1 DLL (33.3° vs. 28.3°, *p*=0.254) were not significantly different between groups (Table 1).

Postoperatively, “aligned” patients showed a significantly higher improvement of LL, TK, and SVA (Table 2).

With regards to postoperative LL distribution, the L1-L4 PLL was not significantly different between groups (18° vs. 16° *p* > 0.05), but the L4-S1 DLL was significantly higher in the “aligned” group (31° vs. 22°, *p*=0.003).

The PI-LL mismatch was significantly correlated with the L4-S1 DLL (rho = 0.407, *p* < 0.001) but not with the L1-L4 PLL (rho = 0.110, *p*=0.342). This significant correlation revealed that restoration of the PI-LL mismatch was significantly related to the correction of the distal lumbar lordosis.

### 3.4. Postoperative Complications and Clinical Evaluation

During the follow-up period, 7 postoperative infections were noted (4 in the “not aligned” group and 3 in the “aligned” group) that required surgical debridement and adapted antibiotics.

At last follow-up, 1 patient out of 35 (3%) in the aligned group had a mechanical complication (rod breakage) that required revision surgery. Six patients out of 42 (14%) of the “not aligned” group had a mechanical complication that required revision surgery (4 rod breakage and 2 proximal junctional failure).

With regards to final follow-up clinical scores, patients from the “aligned” group had a significantly lower lumbar VAS (2.8/10 vs. 4.8/10, *p*=0.028). ODI scores were not significantly different between groups (“aligned” 29% vs. “not aligned” 37%, *p*=0.095).

## 4. Discussion

Surgical management of ASD remains challenging, and optimal management is still under debate. The complication rate after realignment procedures has been reported around 16.5% at 2 years of follow-up and 50% at 10 years of follow-up [10, 11].

According to the literature, a large proportion (up to 50%) of patients remains undercorrected after surgery [7]. Perfect understanding of this undercorrection is difficult especially, as alignment can still change postoperatively. According to McDowell et al. [12], a significant improvement of SVA can be seen during the first postoperative year without further modifications. However, this progressive correction of the SVA was also associated with an increase of pelvic tilt which can be considered as a compensatory mechanism in undercorrected patients.

Restoration of pelvic tilt might therefore be a crucial point for sagittal realignment procedures, especially in patients with a high pelvic incidence. Lafage et al. [13] suggested that patients with a high preoperative pelvic retroversion (related to a high pelvic incidence) require larger lumbar osteotomy procedures. In our series, pelvic tilt was the most difficult parameter to restore, with a nonsignificant postoperative correction. This lack of correction can be related to an insufficient bone resection, but it can also be associated with the level of the osteotomy. Mainly, L3 grade 3 osteotomies have been performed in our experience, and in order to achieve a proper spinopelvic alignment, performing osteotomy at a lower lumbar level (L4 or L5) can increase pelvic tilt correction with an average of 2° per level [14, 15].

Another important parameter for realignment procedures is the postoperative location of the LL apex. Based on Roussouly classification, Pizones et al. recently reported a decrease in mechanical complications with an adapted postoperative lumbar apex position and lumbar shape restoration [16, 17]. Of note, besides sagittal alignment goals, the use of multiple rods constructs has also been reported as a safe and effective method to reduce implant failures [18, 19], but this strategy was not used in our experience.

In our study, the choice was made to stratify patients according to postoperative PI-LL mismatch. Our results revealed that postoperatively “aligned” patients had a preservation of L4-S1 DLL and a higher pelvic incidence when compared to “not aligned” patients. These results are consistent with the study of Ylgor et al. who suggested taking into account lumbar lordosis distribution [20]. While L4-S1 DLL represents around 2/3 of global lordosis, it has been reported that the amount of this L4-S1 DLL was constant and independent from pelvic incidence. However, pelvic incidence is significantly correlated to the L1-L4 PLL [8]. As a whole, these results suggest that the loss of lumbar lordosis mainly occurs in the L4-S1 DLL. As a consequence, failure to restore DLL is correlated with an increased risk of proximal junctional failure [21], and DLL should be preserved or restored around 36° [8].

Results of this study confirm that restoration of L4-S1 DLL is a crucial objective for ASD patients as recently reported by Lafage et al. [22] as 1° of L4-S1 DLL correction produces 10 mm change in SVA and 0.5° in PT. Two strategies can therefore be advocated to reach this goal. First option is based on an anterior approach with intersomatic lordotic cages prior to the posterior fixation. According to recent studies [23–25], this technique can eliminate the need for a grade 3 osteotomy during the posterior approach, especially in primary cases. The second option is to perform L4 or even L5 grade 3 osteotomy [11].

This study presents several limitations such as the one-year follow-up and a limited number of patients included. Further studies will be needed to confirm these results.

## 5. Conclusion

Adult spinal deformity is a frequent and challenging condition for spine physicians. Based on postoperative PI-LL mismatch, the results of this study revealed that maintenance or restoration of L4-S1 DLL is crucial for postoperative alignment. Specific attention must be paid to restore optimal distal lumbar lordosis in order to decrease the rate of undercorrected patients and to improve outcomes.

## Figures and Tables

**Table 1:** Preoperative values of radiographic parameters between “aligned” and “not aligned” groups.

Preoperative	Aligned	Not aligned	*p* value
Pelvic incidence (°)	52	61	0.009
Pelvic tilt (°)	26	33	0.001
L1-S1 lumbar lordosis (°)	34	23	0.004
L1-L4 PLL (°)	1.5	0.8	0.9
L4-S1 DLL (°)	33.3	28.3	0.254
PI-LL (°)	16	34	<0.001
T4-T12 thoracic kyphosis (°)	37	25	0.008
Sagittal vertical axis (mm)	71	91	>0.05

**Table 2:** Postoperative values of radiographic parameters between “aligned” and “not aligned” groups.

Postoperative	Aligned	Not aligned	*p* value
Pelvic incidence (°)	52	61	0.009
Pelvic tilt (°)	24	33	<0.001
L1-S1 lumbar lordosis (°)	49	37	<0.001
L1-L4 PLL (°)	18	16	0.39
L4-S1 DLL (°)	31	22	0.003
PI-LL (°)	4	23	<0.001
T4-T12 thoracic kyphosis (°)	48	40	0.002
Sagittal vertical axis (mm)	23	63	<0.001

## Data Availability

The data used to support the findings of this study are available from the corresponding author upon request.
